# Access to Network Login by Three-Factor Authentication for Effective Information Security

**DOI:** 10.1155/2016/6105053

**Published:** 2016-02-24

**Authors:** S. Vaithyasubramanian, A. Christy, D. Saravanan

**Affiliations:** ^1^Research Scholar, Sathyabama University, Chennai 600119, India; ^2^Research Supervisor, Sathyabama University, Chennai 600119, India; ^3^Faculty of Operations & Systems, IBS, Hyderabad 501203, India

## Abstract

Today's technology development in the field of computer along with internet of things made huge difference in the transformation of our lives. Basic computer framework and web client need to make significant login signify getting to mail, long range interpersonal communication, internet keeping money, booking tickets, perusing online daily papers, and so forth. The login user name and secret key mapping validate if the logging user is the intended client. Secret key is assumed an indispensable part in security. The objective of MFA is to make a layered safeguard and make it more troublesome for an unauthenticated entity to get to an objective, for example, a physical area, processing gadget, system, or database. In the event that one element is bargained or broken, the assailant still has two more boundaries to rupture before effectively breaking into the objective. An endeavor has been made by utilizing three variable types of authentication. In this way managing additional secret key includes an additional layer of security.

## 1. Introduction

In the present computerized day with wonderful improvement in computer segment, single element verification, for example, passwords, is no more analyzed as secure in the World Wide Web. It has never been less troublesome in securing the system and network access. Basic, clear, and simple-to-figure passwords, for example, names and age, are easily discovered through electronic mystery key social occasion programs [1, 2]. The security and protection dangers through malware are dependably contentious both in amount and in quality. Extended access to data builds shortcoming to hacking, splitting of passwords, and online cheats. In this affiliation the routine login/password confirmation is considered insufficiently secure for a few security-basic applications, for example, login to mailing accounts, social networks, gadgets, financial records, authority secured systems, and business sites online. Obliging more than one free element builds the trouble of giving false accreditations [3].

To take care of the password issue in banking sectors furthermore for online exchange two element confirmations utilizing OTP and ATM pin/cards have been actualized. Two-element verification proposition ensures a higher assurance level by augmenting the single confirmation component [4, 5]. Today security concerns are on the rising in all ranges. Most frameworks today depend on static passwords to confirm the client's individuality. Clients have an affinity to utilize evident passwords, basic secret key, effortlessly guessable watchword and the same secret key for various records, and even compose their passwords, store them on their system, or approach the sites for recalling their password, and so forth. Usage of static passwords in this extended reliance on access to IT frameworks logically introduces themselves to hackers, ID thieves, and fraudsters [6]. Furthermore, programmers have the inclination of utilizing various methods/attacks, for example, speculating attack, shoulder surfing attack, lexicon attack, snooping attack, and social designing attack to take passwords in order to obtain entrance to their login accounts [7–9]. A significant number of methods, systems for utilizing passwords have been proposed; however some of which are particularly difficult to utilize and rehearse.

By definition, validation is the utilization of one or more components to demonstrate that you are who you claim to be. Once the personality of the human or machine is accepted, access is conceded. Three all around perceived validation components exist today: what you know (e.g., passwords), what you have (e.g., ATM card or tokens), and what you are (e.g., biometrics). Without supplanting the current validation system, MFA serves as an included layer of security which ensures and improves the current confirmation system [10].

Three-component validation (3FA) is a data security process in which three methods for recognizable proof are consolidated to expand the likelihood that a substance, generally a PC client, is the substantial holder of that personality. 3FA requires the utilization of three solid confirmation elements: something the client (i) knows, for example, an alphanumeric password, (ii) clicks, for example, a graphical password, or (iii) has, for example, unique identity, unique mark, and retinal scan. In this paper an approach has been proposed to enhance the security where a user has to expose their remembering ability by recollecting three factors for their login access. The method proposed is described by schematic algorithm, architecture, and Pseudocode 1. The features, advantages, and limitations are also discussed (see Table 2).

## 2. Existing Authentication Method

The different existing verification to web login is traditional alphanumeric password or graphical password or Biometric Authentication. Alphanumeric password is as a mystery word, an expression, or mix of incidental characters and numbers that validates the personality of the client. Alphanumeric passwords are customary and conventional methods for verification. The human propensity in making secret word makes them helpless and they are liable to different digital attacks. Passwords created with minimum effort and ease of guess are vulnerable to get cracked [6, 11]. CFG password, Markov password, and Array type passwords are innovation in the alphanumeric password [12–15]. In late 1970 biometric frameworks were started. Biometric Authentication depends on the acknowledgment of some physical normality for the identification of the user [16]. One of a kind acknowledgment of the users like face distinguishing proof, voice acknowledgment, iris acknowledgment, and finger print are utilized as a biometric security framework to recognize a verified client. Biometric verification has its own particular quality and confinements. Significant issues in biometric verification are false dismissing rate, false acknowledgment rate, inability to catch, and select rate [17, 18]. In late 1996 validation utilizing graphical secret key appeared. The client can pick a pass point or predefined areas in a picture as their secret word. Graphical secret key methodologies are additionally called graphical password [19]. Graphical passwords are liable to different attacks like edge identification strategies, shoulder surfing, and so forth [20–23]. In this digital world, passwords play a crucial role in enhancing the data security.

## 3. Proposed Authentication Method

### 3.1. System Design

Keeping up security is turning out to be more testing with time. A percentage of the difficulties can be foreseen; for example, propels in calculation that are making it continuously less demanding to word reference attack a secret key database. Different difficulties are harder to suspect, for example, the revelation of new “day-zero” vulnerabilities in working programming. Hence, security prerequisites are not altered, but rather increment with time. Multifactor confirmation is regularly being utilized to work around the basic shortcomings in secret key administration. While three-element verification improves security, it expands client grating, a specific issue for online administrations that are not in a position to command 3FA. Incorporated 3FA gives the best ease of use to better security, so a three-component verification innovation that can be moved up to coordinate the three variables all the more nearly has the best capacity to develop as requirements change and additionally to boost client uptake of discretionary 3FA.

After the client gives their username, three methods of operation are accessible for the clients in light of their preference and requirements. The main is a stand-alone approach that is anything but difficult to utilize and secure and is traditional. The second approach is picture-based methodology that is likewise simple to utilize and secure yet requires system designs and the third approach is biometric verification which is something the client has like unique finger impression, palm print, and retinal output, yet turns costly.

*System Design.* System design is as follows:

 Start;
 Login ID; user ID;
 First factor: alphanumeric password;
 Authentication approval: admin;
 If authentication fails alarm the user/if authentication is accepted proceed for second gate way;
 Second factor: graphical password;
 Authentication approval: admin;
 If authentication fails alarm the user/if authentication is accepted proceed for third gate way;
 Third factor: biometric password;
 If authentication fails alarm the user/if authentication is accepted access the account;
 Login authorization.

### 3.2. Implementation Process

Nowadays service providers across World Wide Web applications insist that users make their own particular login account for better administration of their user database and transaction tracking. A typical system user exercises multiple login accounts with various frequency of usage. Many users tend to keep the same password for many login accounts for better recollection of login ID and password data. To make it simple the clients use various techniques as password administration tool, store them in mail account, use framework to recollect their secret key, use bit of paper by composing their password, and sometimes share their password.

Pitfalls associated with creating self-style login password lead to easier hacking of user accounts. Users can categorize their login accounts as business critical, high risk, and low risk to protect their confidentiality and security (see Table 1).

To effectively challenge the risks associated with the security threats on password, the web administrator or service provider can offer the options of password method to client. While creating login account, the client ought to be furnished with his choice of one variable or three-component verification by administration supplier. However based on the account category the administrator can provide suggested method as a recommendation to the user but freeze user's selection of password method as final. Administrator can specify their restrictions on allowing change of password method if any, after account creation. Once client chooses his password method as 3-factor or single factor and saves, depending on his selection, the password generation should be processed and handled. The suggested 3-factor authentication would also provide the clients cross browser compatibility (consistency in web components across different browsers like Explorer, Firefox, Chrome, Opera, etc.) which would be a key requirement for business and personal data confidentiality and integrity.

## 4. Features, Advantage, and Disadvantage of 3FA

Multifaceted confirmation is a security system in which more than one sign of affirmation is executed to affirm the genuineness of a trade. The procedure that demands various reactions to test request and recuperates “something you have” or “something you are” is considered multifaceted. Multifaceted affirmation is a security structure in which more than one appearance of assertion is executed to insist on the validness of an exchange. Three component affirmations in like manner have confinements which fuse the cost of purchasing, issuing, and managing it. Requiring more than one autonomous variable builds the trouble of giving false accreditations. Despite the fact that it is not less demanding to utilize and shoddy it is rather secure. The passwords are client picked not given by whatever other password administration frameworks and furthermore kept up by administration supplier not by password administration system.

## 5. Conclusion

In redesigning the authentication service providers and users have to look into future verification necessities, not today's. As a rule, one needs to spend more to get more elevated amounts of security. Three-element confirmation arrangement prepares clients by giving adaptable and solid validation to expansive scale. Three element validation frameworks are easy to use approach and require memorability of verification passwords. The objective of security to keep up the trustworthiness, accessibility, and protection of the data endowed to the framework can be gotten by adjusting this verification method. Three-factor authentication (3FA) could definitely diminish the frequency of online extensive fraud and other online extortions, in light of the fact that the victims password would never again be sufficient to give a hoodlum access to their data.

## Figures and Tables

**Pseudocode 1:**
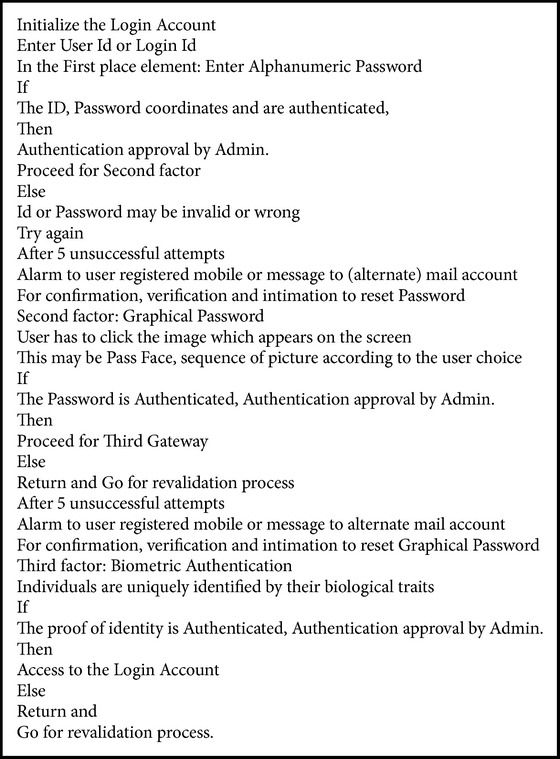
Three-factor authentication.

**Table 1:** 

Account category	Description/example	Damages by security threats or failure	Suggested password method
Business critical	Business emails with client data	Business, strategic and employee confidentiality; brand image, reputation	Extremely secure: 3-factor authentication
High risk	Financial like bank acct, online purchases, and personal email	Financial data exposure, monetary loss, and personal information exposure	Highly secure: 3-factor authentication
Low risk	Social networking and enquiry sites	Identity exposure	Moderate: 1 factor authentication

**Table 2:** 

Advantages	Disadvantages
Information security	Time consuming
Secured login: secures websites, portals, and web applications	Space complexity
Depth in defense: three levels of protection	Remembering ability
Trust ability towards the service provider by the users	System configuration
